# Interesting Cases

**Published:** 1848

**Authors:** James Blake

**Affiliations:** Professor of Anatomy in the St. Louis University


ARTICLE III.

*Interesting Cases by* JAMES BLAKE, M.D., Professor of Anatomy in the St. Louis University — *Abnormal distribution of the Thyroid Arteries.*

The following case of abnormal distribution of the thyroid arteries, is, I think, of sufficient interest to render it worthy of the attention of the profession; as, in the subject in which it occurred, neither in the operation of tracheotomy or laryngotomy could have been performed without great danger of sacrificing the life of the individual. The subject in which it occurred was a male about forty years of age, brought into the dissecting room of the St. Louis University during the past session. The first anomaly that presented itself on dissecting the arteries going to the head and neck, was of a considerable artery about the size of a quill, arising from the anterior and superior of the innomminata, passing upwards and crossing the trachea about three-quarters of an inch above the sternum. After proceeding about two lines beyond the mesial line, it again turned to the right and crossed the mesial line about a quarter of an inch before the isthmus of the thyroid body, and proceeded to divide into branches along its lower edge on the right side.

On dissecting the superior thyroid arteries, it was found that the artery of the right side was subject to an abnormal distribution, perhaps more dangerous to the operator than was the artery coming from the innominata; for whilst the latter has been noticed as not of very unfrequent occurrence, this abnormal distribution of the superior thyroid must, I think, be extremely rare, as I see no notice of it in those anatomical and surgical works which I have referred to. The artery arose from the external carotid, at the place where it is generally found; but here it was seen to be much larger than usual; it proceeded downwards to the upper and outer angle of the thyroid body, but instead of dividing into its terminal branches, as it generally does, it turned forwards to the left, running along the upper edge of the cricoid cartilage, or between it and the thyroid cartilage, and lying on the crico-thyroid muscle; it continued this course until it passed rather beyond the mesial line, crossing the crico-thyroid ligament. During its course, it sent branches downwards to the upper edge of the right side of the thyroid boby, and the isthmus and its terminal branches were distributed to the left lake of the thyroid body. The artery where it crossed the crico-thyroid ligament was as large as a crow quill; there was no large anastomiotic branch uniting with the superior thyroid artery of the opposite side, which was rather smaller than natural. The inferior thyroid arteries were natural, but that of the right side rather larger. The thyroid body was rather above the natural size, and somewhat more dense in its structure, and less red than is generally found—the isthmus was broad, extending as far as the lower edge of the cricoid cartilage. From the above description it is evident that neither the operation of tracheatomy or laryngotomy could have been performed in this subject without incurring the greatest risk of wounding an artery, the bleeding from which might under the circumstances, even had led to a fatal result.

*Case of Entozoa in the heart.*—On opening the heart of a dog which had been the subject of a physiological experiment, the right auricle was found to contain a number of entozoa, between twenty or thirty, they were situated principally in the auriclar appendage, forming an intricate mass. The worms appeared to be a species of ascaris, they were smaller in character than the ascaris lumbricoides, and varied in length from four to eight inches—they were so completely twisted one with the other, as to form an intricate mass, which more than filled the anicular appendage. The animal did not appear to have at all suffered from the presence of these parasites, for it seemed perfectly healthy. The occurrence of entozoa in the heart has, I find, been noticed by two or three writers, but it would appear that their occurrence is extremely rare. Besides the interesting question, as to how these animals would find their way into their extraordinary habitation, another still more serious appears to me to be, how they became enveloped in a part which is constantly being washed out as it were by the blood, and from which, it would seem, that they must be expelled, as each contraction of the ventricle propelled forwards the fluid in which they existed. I regret that in consequence of the preparation having been destroyed by the rats, I was unable to examine it as minutely as I could have wished.—*St. Louis Med. and Surg. Jour.*

